# The DNA damage checkpoint response to replication stress: A Game of Forks

**DOI:** 10.3389/fgene.2013.00026

**Published:** 2013-03-13

**Authors:** Rachel Jossen, Rodrigo Bermejo

**Affiliations:** Instituto de Biología Funcional y Genómica, CSIC/USALSalamanca, Spain

**Keywords:** replication forks, DNA damage checkpoint, genomic instability, Mec1/ATR, oncogene stress

## Abstract

Conditions challenging replication fork progression, collectively referred to as replication stress, represent a major source of genomic instability and are associated to cancer onset. The replication checkpoint, a specialized branch of the DNA damage checkpoint, monitors fork problems, and triggers a cellular response aimed at preserving genome integrity. Here, we review the mechanisms by which the replication checkpoint monitors and responds to replication stress, focusing on the checkpoint-mediated pathways contributing to protect replication fork integrity. We discuss how cells achieve checkpoint signaling inactivation once replication stress is overcome and how a failure to timely revert checkpoint-mediated changes in cellular physiology might impact on replication dynamics and genome integrity. We also highlight the checkpoint function as an anti-cancer barrier preventing cells malignant transformation following oncogene-induced replication stress.

## Introduction

During S phase, cells must faithfully duplicate their genomes. For this purpose eukaryotic cells establish multiple replication forks, specialized structures where DNA synthesis is carried out, that traverse the entire genome in a coordinated manner, thus granting a timely chromosomal replication (Bell and Dutta, 2002). Replication forks are complex structures in which parental DNA is unwound to produce a single-stranded DNA (ssDNA) template for replicative DNA polymerases (Johnson and O'Donnell, 2005). Due to the presence of ssDNA and the necessity to finely tune the functions of the diverse replisome components, replication forks are fragile structures prone to accumulating DNA breaks and being engaged by recombinational repair machineries (Branzei and Foiani, 2010). Conditions that impair DNA synthesis at replication forks or interfere with their progression, collectively termed “replication stress”, can alter replication fork structure and functionality thus priming chromosomal breakage and unscheduled recombination events. Recent evidence has suggested that replication stress can be a major source of spontaneous genomic instability driving malignant transformation of pre-cancerous cells (Bartek et al., 2007b).

Eukaryotic cells have evolved mechanisms, usually termed as the replication checkpoint, that monitor the occurrence of replication stress and trigger a cellular response aimed at preserving genome integrity. The replication checkpoint constitutes a specialized branch of the DNA damage checkpoint and it is often referred to as the S phase (or intra-S phase) checkpoint (Paulovich and Hartwell, 1995; Boddy and Russell, 2001; Nyberg et al., 2002; Osborn et al., 2002). Even if it was originally described as a signal transduction pathway delaying cell cycle progression to provide time to allow replication to finish (Enoch and Nurse, 1990; al-Khodairy and Carr, 1992; Enoch et al., 1992; Rowley et al., 1992; Weinert, 1992), work over the last 25 years has revealed that the replication checkpoint is a complex response with highly interconnected players, which regulates an unprecedented variety of cellular processes in order to sustain cell viability and protect genome integrity (Branzei and Foiani, 2009; Segurado and Tercero, 2009; Zegerman and Diffley, 2009, 2010; Labib and De Piccoli, 2011). Here we will review the current understanding of how the replication checkpoint senses and responds to replication stress, based mainly on the work carried out in the budding yeast model system. We will discuss recent evidence that sheds light on the checkpoint's essential function in promoting replication fork stability and on how cells inactivate checkpoint signaling to restore normal cell physiology. We will also consider the checkpoint from an evolutionary perspective and illustrate how it might act to suppress unrestrained proliferation and tumor progression in multicellular organisms.

## Replication fork stalling and checkpoint signaling

Eukaryotic cells establish multiple replication forks in a time-regulated fashion due to the orderly activation of replication origins throughout S phase (Raghuraman et al., 2001). The Mcm2–7 complex replicative helicase unwinds the parental DNA helix, thus generating a ssDNA template for the replicative polymerases (Waga and Stillman, 1998). RPA stabilizes ssDNA tracks facilitating DNA synthesis and suppressing their engagement by recombination factors (Iftode et al., 1999). DNA polymerase ε is thought to carry out leading strand synthesis, while DNA polymerase α and DNA polymerase δ primarily synthesize the lagging strand (Pursell et al., 2007). The replication fork is a complex structure in which DNA synthesis is coordinated with other DNA metabolic processes. A number of additional factors associate with replication forks to assist DNA polymerases processivity, lagging strand maturation, topological stress simplification, replisome stabilization, and coordination between replication and sister chromatid cohesion establishment (Tourriere et al., 2005; Gambus et al., 2006; Lengronne et al., 2006; Bermejo et al., 2007; Moldovan et al., 2007).

Faithfull DNA replication requires that replication forks are processive and stable so that DNA synthesis is carried out with high fidelity throughout the genome. Replication fork progression can stall due to different causes. Template unwinding by replicative helicases can be counteracted by topological constraints, higher order DNA structures, or tightly DNA bound proteins (Azvolinsky et al., 2006; Bermejo et al., 2007; Labib and Hodgson, 2007). Additionally, damaged DNA and DNA synthesis inhibition owing to endogenous or exogenous factors may hamper replication fork progression. Agents generating DNA-topoisomerase adducts, intra-strand crosslinks or bulky DNA adducts can block the action of replicative helicases, whilst the progression of DNA polymerases can be impaired by the presence of base-adducts (as the ones generated by methylmethansulphonate—MMS) or by direct inhibition of DNA synthesis (for instance through the depletion of dNTP pools induced by hydroxyurea) (Branzei and Foiani, 2010; Zegerman and Diffley, 2010; Ray Chaudhuri et al., 2012). Replication forks can also interfere with other DNA metabolism machineries. DNA and RNA polymerases compete for the same template during S phase and indeed replication machinery interference with the transcriptional apparatus has emerged as a major cause of fork collapse (for a recent review see Bermejo et al., 2012b). The mechanisms determining replication interference with transcription are not fully understood, though they might implicate clashes between replicative helicases and transcriptional machineries, topological interference with higher order chromatin structures established by co-transcriptional processes [such as gene loops or association with nuclear pore complexes (NPCs)] or engagement of aberrant RNA:DNA hybrids formed by the annealing of nascent RNAs (Deshpande and Newlon, 1996; Azvolinsky et al., 2009; Bermejo et al., 2009; Gomez-Gonzalez et al., 2011; Alzu et al., 2012).

When replication forks stall, a signaling cascade mediated by DNA damage checkpoint kinases is activated, spreading checkpoint signaling to a number of effectors that regulate diverse aspects of cell physiology. Factors involved in sensing and transducing the checkpoint signals generated at replication forks are highly conserved amongst eukaryotes (Table 1). Unless otherwise stated, we will refer in this review to the budding yeast homologs of these factors. At the center of the checkpoint signaling cascade are the phosphoinositide 3-kinases (PI3)-related Mec1 (HsATR) and Tel1 (HsATM) kinases (Weinert et al., 1994; Greenwell et al., 1995; Morrow et al., 1995; Savitsky et al., 1995; Bentley et al., 1996; Mallory and Petes, 2000; Paciotti et al., 2001). Human ATR and ATM are important to suppress malignant transformation and can be found mutated in cancer cells (Kastan and Bartek, 2004). The highly conserved effector kinases Rad53 and Chk1 are directly targeted by PI3-related kinases and are responsible for the amplification of the checkpoint signal, as well as for the phosphorylation of key proteins that modulate different aspects of cellular physiology (Longhese et al., 2003).

**Table 1:** **Replication checkpoint sensors and transducers**.

**Function**	***S. cerevisiae***	***S. pombe***	***H. sapiens***
Sensors	Rfa1	Ssb1	RPA70
	Rfa2	Ssb2	RPA32
	Rfa3	Ssb3	RPA14
Apical kinases and interacting proteins	Mec1	Rad3	ATR
	Tel1	Tel1	ATM
	Ddc2	Rad26	ATRIP
Transducers	Mrc1	Mrc1	CLASPIN
	Rad9	Crb2	–
Effector kinases	Rad53	Cds1	CHK2
	Chk1	Chk1	CHK1
	Dun1	–	–

**Figure 1:**
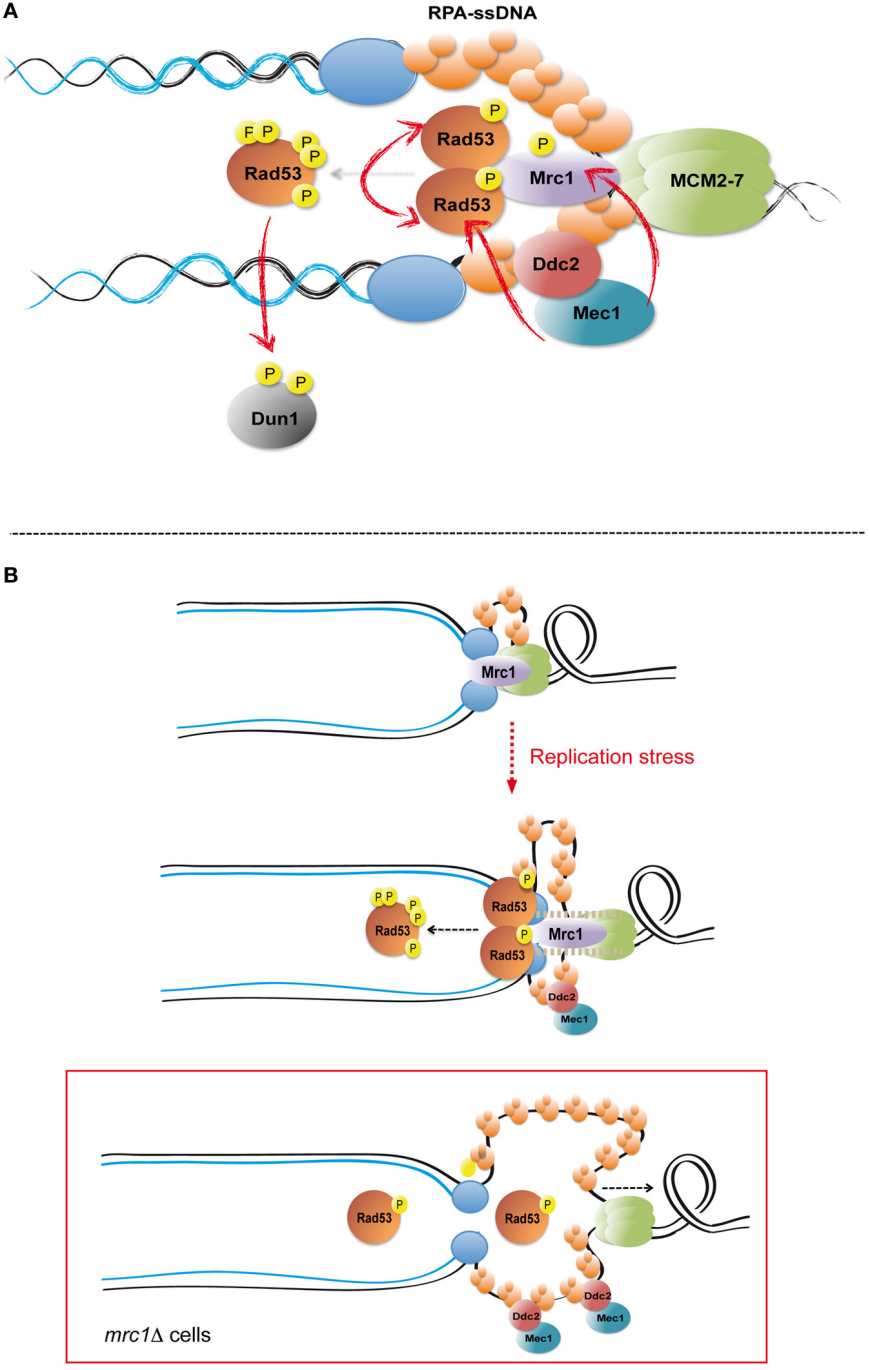
**Checkpoint activation in response to replication stress.** **(A)** Upon replication fork stalling ssDNA is generated by the replicative helicase—DNA polymerases uncoupling. RPA-ssDNA mediates the recruitment of the apical checkpoint kinase Mec1 to replication forks by the action of its associated factor Ddc2. Mec1 phosphorylates fork components, including the Mrc1 transducer, and the Rad53 effector kinase. Mrc1 serves as a scaffold promoting Rad53 trans-autophosphorylation events and full kinase activation. Rad53 phosphorylates and activates the Dun1 effector kinase. Red arrows indicate key phosphorylation events mediating checkpoint activation. **(B)** Mrc1 is a replisome component that travels with replication forks in unperturbed conditions. Following replication stress, Mrc1 prevents excessive DNA unwinding by restraining Mcm2–7 helicase progression, likely by physically tethering DNA helicases and polymerases. The tension generated between stalled polymerases and advancing helicases may determine conformational changes in Mrc1, thus promoting its function as a molecular scaffold necessary for Rad53 trans-autophosphorylation reactions. In Mrc1 ablated cells (*mrc1*Δ), failure to restrain Mcm2–7 helicase leads to extensive DNA unwinding and ssDNA accumulation at replication forks, which does not directly result in Rad53 hyper-phosphorylation and full kinase activation due to the absence of Mrc1-mediated scaffolding.

Checkpoint activation in response to replication stress requires the presence of replication forks (Lupardus et al., 2002; Stokes et al., 2002). Indeed, it is thought that the generation of extended ssDNA tracks at replication forks is the main signal triggering replication checkpoint activation (You et al., 2002; Zou and Elledge, 2003). ssDNA at forks can be generated in response to replication inhibitors (such as hydroxyurea or aphidicolin), due to the uncoupling between DNA unwinding by helicases and the progression of DNA polymerases (Sogo et al., 2002; Byun et al., 2005), or by the uncoupling between leading and lagging strand polymerases due to the presence of damaged templates (Branzei and Foiani, 2009). Extended ssDNA tracks are readily coated by the single strand DNA-binding protein RPA complex (composed of Rfa1, Rfa2, and Rfa3) (Zou and Elledge, 2003) (Figure 1A), which recruits the apical kinase Mec1 to stalled forks through the action of its associated factor Ddc2 (Zou and Elledge, 2003). Upon recruitment to fork DNA, Mec1 phosphorylates several factors including Mrc1 (Alcasabas et al., 2001; Tanaka and Russell, 2001). Mrc1 is a structural component of the replication fork required for both DNA replication and checkpoint signaling (Osborn and Elledge, 2003; Szyjka et al., 2005; Tourriere et al., 2005). In response to replication stress Mrc1 acts as a signal transducer mediating full Rad53 kinase activation (Alcasabas et al., 2001). By analogy with the paradigmatic checkpoint transducer Rad9, Mrc1 is thought to act as a scaffold promoting Rad53 trans-autophosphorylation events (Pellicioli and Foiani, 2005; Chen and Zhou, 2009; Berens and Toczyski, 2012). Mec1 phosphorylates Rad53 in a Mrc1-dependent manner, and full kinase activity is achieved when different Rad53 molecules hyperphosphorylate each other before being released to reach their targets (Pellicioli and Foiani, 2005).

Mrc1 is necessary to sustain normal fork progression rates in the absence of genotoxic stimuli (Tourriere et al., 2005). In addition, Mrc1 prevents extensive uncoupling between helicase unwinding and DNA synthesis at stalled forks by somehow tethering helicases to DNA polymerases (Katou et al., 2003; Nedelcheva-Veleva et al., 2006). Importantly, Mrc1 interacts with polymerase ε catalytic subunit Pol2 in a checkpoint-dependent manner (Lou et al., 2008). Hence Mrc1 might act as a “molecular spring” sensing the physical connection between helicases and polymerases and at the same time preventing their uncoupling (Figure 1B). In this view, Mrc1 might suppress futile checkpoint signal amplification in forks to which Mec1 is recruited to ssDNA but the uncoupling between helicases and polymerases cannot be “physically” sensed.

Modulation of cellular physiology in response to replication stress is ultimately achieved through the regulation of a variety of effectors, which is mediated by phosphorylation events carried out by Mec1, Rad53, and Dun1 kinases (Table 2). Mec1 is thought to act locally by phosphorylating replication fork-associated (Smolka et al., 2007; Randell et al., 2010) and chromatin factors (Randell et al., 2010; Rodriguez and Tsukiyama, 2013). Several Rad53 targets are instead not localized at forks (Smolka et al., 2007; Chen et al., 2010), consistent with the notion that Rad53 may diffuse and propagate checkpoint signaling to distant effectors throughout the nucleus. Recently, the importance of checkpoint-mediated regulation of NPC and nuclear membrane-related processes in genome integrity maintenance has been revealed (Bermejo et al., 2011, 2012a) and it has been proposed that Rad53 might also regulate processes taking place in the cytoplasm (Enserink et al., 2006). Rad53 targets include the Dun1 kinase (Bashkirov et al., 2003), partially related to Rad53 (Zhou and Elledge, 1993; Rhind and Russell, 1998), which promotes the transcriptional induction of damage inducible genes and dNTP pool upregulation. The checkpoint response was originally considered a canonical signal transduction cascade composed by upstream sensors and a number of signal transducer kinases that regulate a large number of downstream effectors (Longhese et al., 2006). The picture though seems far more complex as factors can exert different roles in the cascade. For instance, Mec1 acts as a sensor and a signal transducer, but also directly phosphorylates effector proteins. Additionally, several sensors and transducers (as RPA complex proteins, Ddc2, or Mrc1) are directly phosphorylated by checkpoint kinases. Below we describe the better-characterized checkpoint-regulated processes contributing to maintain replication fidelity and genome integrity.

**Table 2:** **Checkpoint kinases phosphorylation targets overview**.

**Checkpoint kinase**	**Regulated process**	**Targets**
Mec1	DNA replication	Cdc2, Dpb4, Mcm4, Pol31, Psf1, Rfa1, Rfa2
	Checkpoint response	Ddc2, Dun1, Mec1, Mec3, Mrc1, Rad9, Rad17, Rad53
	DNA repair	Mlh1, Msh6, Rad23, Rad26, Rad55, Rtt107, Sae2, Slx4
	dNTP pools regulation	Ssn6
	Chromatin structure	Abf1, Hta1, Ies4, Isw2, Sin3, Sir4, Swi3
	NPC function	Hpr1, Nup2, Nup60
	Other	Cbf1, Cdc13, Nma111, Rif1, Spt7, Sum1
Rad53	DNA replication	Sld3, Rad27, Dbf4, Ctf4, Pol1
	Checkpoint response	Ddc1, Ddc2, Dun1, Mrc1, Rad9, Rad53, Tof1
	DNA repair	Exo1, Rad54, Rad55, Rtt107
	dNTP pools regulation	Crt1, Nrm1, Rnr3, Swi6
	Chromatin structure	Hhf1, Hho1, Hpc2, Esc1, Fun30, Itc1, Rph1, Snf2
	NPC function	Mlp1, Nsp1, Nup1, Nup2, Nup60, Hpr1
	Other	Mcd1, Plm2, Ycg1
Dun1	Checkpoint response	Dun1
	DNA repair	Nej1
	dNTP pool regulation	Crt1, Dif1, Rnr3, Sml1
	Chromatin structure	Hpc2, Rco1
	NPC function	Mlp1, Nup159
	Other	Ecm21, Npl3, Sec3

## Checkpoint control of S phase transcription and dNTP pools

The checkpoint response modulates cellular physiology to promote cell survival and preserve genome integrity. One of the earliest described checkpoint functions in response to replication stress is the delay of the progression through mitosis, which is achieved through direct modification of key cell cycle regulators and prevents the premature segregation of incompletely replicated chromosomes (Krishnan et al., 2004; Putnam et al., 2009; Palou et al., 2010). However, the majority of checkpoint-regulated events relevant for cell viability and genome integrity maintenance are thought to take place in S phase, including upregulation of dNTP pools, inhibition of origin firing, stabilization of replication forks, and modulation of DNA repair. The functional meaning of other checkpoint-mediated effects such as modulation of transfer-RNA (tRNA) genes metabolism (Ghavidel et al., 2007; Nguyen et al., 2010) or the cellular redox state (Carter et al., 2005) are less clear, though repression of tRNA genes might counteract fork collapse by preventing forks clashing with the transcriptional apparatus (Nguyen et al., 2010).

**Figure 2:**
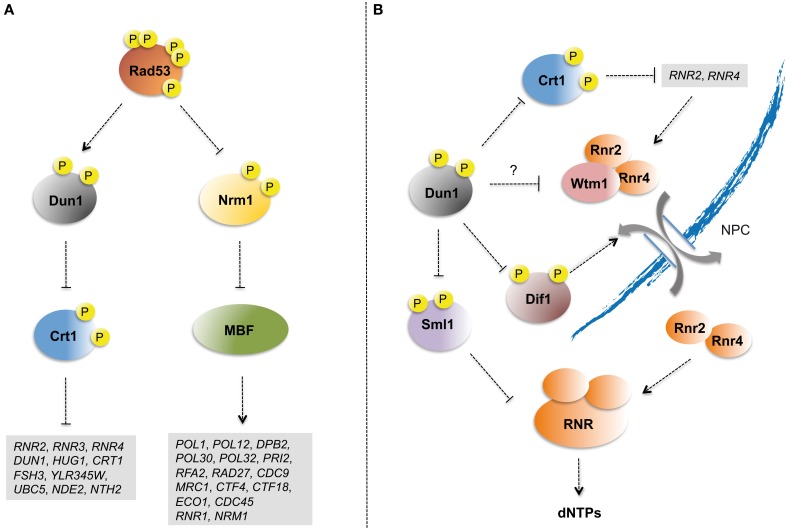
**Checkpoint control of S phase transcription and dNTP pools. (A)** Rad53 kinase controls the transcriptional activation of Crt1-repressed damage-inducible and G1/S transition MBF genes in response to replication stress. The transcriptional repressor Crt1 is phosphorylated in a Rad53- and Dun1-dependent manner and displaced from the promoters of damage-inducible genes. Rad53 also phosphorylates the MBF-specific repressor Nrm1 allowing the expression of G1/S transition genes. Relevant genes with roles including dNTP pool regulation, checkpoint response, DNA replication, and DNA repair that are induced following replication stress in a checkpoint-dependent manner are listed. **(B)** Dun1 regulates ribonucleotide reductase activity through multiple mechanisms. Dun1 phosphorylates Sml1, which binds and inhibits RNR catalytic subunit Rnr1, promoting its degradation. Dun1 also phosphorylates and promotes the degradation of Dif1, which mediates Rnr2/Rnr4 subcomplex nuclear import. Rnr2/Rnr4 subcomplex nuclear retention is mediated by its association with Wtm1. Wtm1-Rnr2/Rnr4 interaction is lost upon checkpoint activation through unknown mechanisms that have been proposed to depend on Dun1-mediated phosphorylation. Lastly, Dun1 upregulates the transcription of RNR subunits through phosphorylation and inhibition of Crt1.

Checkpoint kinases modulate the transcriptional program of cells experiencing replication stress (Smolka et al., 2012). The Dun1 kinase upregulates the transcription of damage inducible genes by phosphorylating Crt1 (Huang et al., 1998) (Figure 2A). Crt1 binds to gene promoters and attracts the general transcriptional repressors Ssn6 and Tup1 (Huang et al., 1998). Dun1-dependent phosphorylation displaces Crt1 from promoter chromatin leading to the transcriptional activation of several genes, including *DUN1* itself and genes involved in dNTP synthesis, such as the ribonucleotide reductase (RNR) subunits encoding genes *RNR3*, *RNR2*, and *RNR4* (Zhou and Elledge, 1993; Zaim et al., 2005). Upregulation of *DUN1* expression feeds checkpoint signaling, thus contributing to strengthening Dun1-mediated control of dNTP levels (see below).

The MBF (Mlu1-box Binding Factor) heterodimeric transcription factor drives the expression of a variety of genes required for G1/S transition (Koch et al., 1993). MBF transcription is repressed upon S phase entry through the binding of the MBF-associated Nrm1 co-repressor (de Bruin et al., 2006). Nrm1 is a phosphorylation target of both Rad53 and its fission yeast ortholog Cds1 (de Bruin et al., 2008; Travesa et al., 2012) (Figure 2A). Rad53-mediated Nrm1 phosphorylation prevents it from binding to the MBF promoters, thus leading to transcriptional upregulation of G1/S transition genes (Travesa et al., 2012). Genes whose expression is upregulated by Rad53 and Nrm1 in response to replication stress encode factors directly involved in DNA synthesis (i.e., *RNR1*, *RFA2*, *POL1*, *POL12*, *POL30*, *POL32*, *PRI2*, and *DPB2*), lagging strand maturation (i.e., *CDC9* and *RAD27*), replisome components, and accessory factors (i.e., *MRC1*, *CDC45*, *CTF4*, *CTF18*, *ECO1*, and *ELG1*) (Travesa et al., 2012).

The functional meaning of checkpoint-induced transcription is unclear. Preventing protein synthesis by cycloheximide treatment has little impact on cell survival following replication stress (Tercero et al., 2003). This observation led to the suggestion that checkpoint-induced transcription has a relatively small contribution to the stabilization of stalled replication forks. However, Nrm1 ablation confers resistance to hydroxyurea treatment (de Bruin et al., 2006, 2008) and Crt1 deletion mutants show increased viability following HU or MMS exposure (Shen et al., 2007 and our unpublished observations). Hence the transcriptional upregulation of key factors might be important to promote cell viability, perhaps by contributing to the stabilization of replication forks or the upregulation of dNTP levels. Further investigation will be required to elucidate this intriguing connection.

Tight regulation of dNTP pools is essential for cells to guarantee viability and prevent elevated mutagenesis rates (Chabes et al., 2003). Deregulation of the dNTP pool leads to genomic instability in yeast (Zhao et al., 2001) and mammalian cells (Bester et al., 2011). The replication checkpoint upregulates dNTP levels in response to replication stress, mainly through modulation of RNR activity (Figure 2B). RNR is a multimeric enzyme that catalyzes the reduction of ribonucleotides to deoxyribonucleotides, the rate-limiting step in dNTP synthesis. During most of the cell cycle the large catalytic subunit Rnr1 localizes to the cytoplasm, while Rnr2-Rnr4 subcomplex is nuclear (Yao et al., 2003). Dif1 directly binds and mediates the nuclear import of the Rnr2-Rnr4 subcomplex (Lee et al., 2008), which is retained in the nucleus through the action of Wtm1 (Lee and Elledge, 2006). Activation of checkpoint kinases leads to the re-localization of the RNR small subunits Rnr2-Rnr4 from the nucleus to the cytoplasm (Lee and Elledge, 2006). Following replication stress Dun1 phosphorylates Dif1 inducing its degradation (Lee et al., 2008) and Wtm1 interaction with the Rnr2-Rnr4 complex is abrogated (Lee and Elledge, 2006). Redistribution of Rnr2-Rnr4 to the cytoplasm favors its association with Rnr1 to constitute an active RNR complex and upregulate dNTP levels. As mentioned above, RNR subunits are transcriptionally induced in response to replication stress. A more striking effect is observed for *RNR3*. Rnr3 is an alternative catalytic subunit that can substitute Rnr1 to form active RNR complexes (Domkin et al., 2002) targeted by Rad53 (Smolka et al., 2007). *RNR3* has very low expression levels in the absence of genotoxic stresses and its protein levels following replication stress are relatively low as compared to those of Rnr1 (Li and Reese, 2001; Domkin et al., 2002). Hence, the functional role of Rnr3 in dNTP pool regulation remains unclear.

Checkpoint kinases also upregulate dNTP levels through Sml1, a small protein that directly binds to Rnr1 and inhibits RNR enzymatic activity (Zhao et al., 1998; Chabes et al., 1999). Phosphorylation of Sml1 by Dun1 triggers Sml1 degradation via a complex formed by the E2 ubiquitin-conjugating enzyme Rad6, the E3 ubiquitin ligase Ubr2, and the accessory factor Mub1 (Zhao and Rothstein, 2002; Andreson et al., 2010). Upregulation of dNTP pools could contribute to stabilizing replication forks by directly increasing polymerase processivity or by facilitating a more efficient repair of lesions blocking fork progression. Importantly, Mec1 and Rad53 are thought to regulate dNTP pools in unperturbed S phase, as the lethality of *MEC1* or *RAD53* deletion is suppressed by Sml1 ablation (Zhao et al., 1998), and defective dNTP pool regulation in checkpoint mutants results in spontaneous fragility of hard-to-replicate genomic regions (Cha and Kleckner, 2002).

## Checkpoint control of replicon dynamics and fork stability

Cells experiencing replication stress modulate chromosomal replication through at least two checkpoint-dependent mechanisms: the stabilization of stalled replication forks and the block of origin firing. Replication origins fire with a somewhat pre-defined timing throughout unperturbed S phases (Raghuraman et al., 2001). In response to replication stress origin firing is regulated by checkpoint kinases that mediate the repression of late and dormant origins (Santocanale and Diffley, 1998; Shirahige et al., 1998). This effect is directly mediated by Rad53, which phosphorylates Dbf4 and Sld3 proteins thus short-circuiting the two alternative Dbf4-dependent kinase (DDK) and cyclin-dependent kinase (CDK) pathways that promote origin firing in S phase (Lopez-Mosqueda et al., 2010; Zegerman and Diffley, 2010). Interestingly, dormant origin derepression also takes place when a double strand break (DSB) is induced at a neighboring HO-endonuclease sequence in the budding yeast mating type locus (Doksani et al., 2009). HO-break mediated origin derepression occurs even when Rad53 is fully activated owing to HU treatment. Hence alternative mechanisms, perhaps involving chromatin structure changes, might bypass checkpoint control on origin firing. Prevention of late origin firing in response to replication stress seems to have obvious advantages for the cell. When forks stall due to reduced dNTP levels, establishing more replication forks at late origins would further increase dNTPs demand. In the presence of damaged templates, limiting late origin firing would prevent additional forks to stall by running into DNA lesions. However, the inability to prevent late origin firing is not thought to be the major cause of cell lethality in checkpoint mutants experiencing replication stress as *mec1-100* mutants, which fail to prevent late origin firing, are not sensitive to HU or MMS treatments (Tercero et al., 2003).

The most crucial function exerted by checkpoint kinases is the protection of fork stability (Lopes et al., 2001; Tercero and Diffley, 2001; Sogo et al., 2002), which has been argued to account for the maintenance of cell viability following replication stress (Segurado and Diffley, 2008). In checkpoint mutants, replication forks fail to resume DNA synthesis after removal of replication stress-inducing drugs (Desany et al., 1998) and accumulate DNA breaks (Feng et al., 2006; Raveendranathan et al., 2006; Feng et al., 2011). The loss of replication fork functional integrity accompanied by structural alterations of replication intermediates is usually termed fork collapse and it is thought to be a major cause of gross chromosomal rearrangements in checkpoint-deficient cells (Myung et al., 2001; Myung and Kolodner, 2002; Admire et al., 2006). Fork stability defects also result in an increased incidence of malignant tumors (Kawabata et al., 2011). Currently the checkpoint-mediated mechanisms counteracting fork collapse are not fully understood, though fork-protecting pathways may interplay.

**Figure 3:**
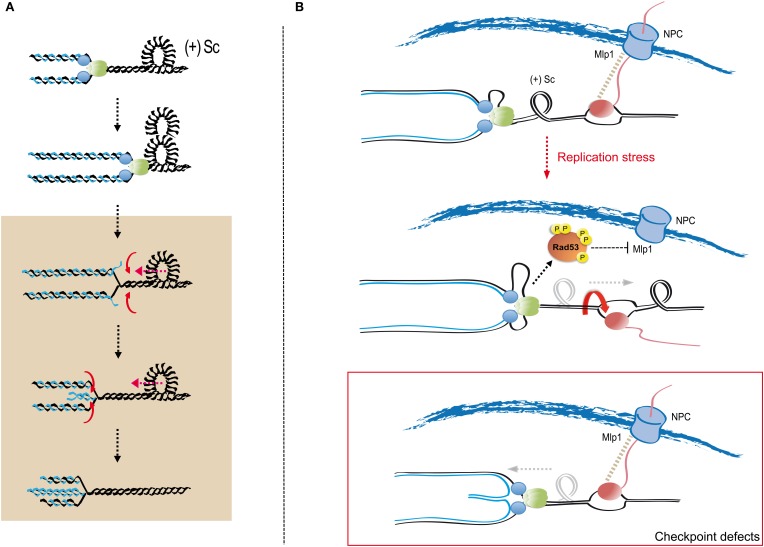
**Replication fork reversal and checkpoint-mediated topological simplification at transcribed regions. (A)** DNA double helix unwinding during replication generates torsional stress that can accommodate as positive supercoiling (+Sc) ahead of replication forks. Progressively accumulating positive supercoiling provides the driving force for replication fork reversal; particularly upon the dissociation of replisome components from fork DNA. Positive supercoiling can be re-accommodated by re-winding of the parental strands, which results in the regression of the fork branching point and the extrusion of newly synthesized strands (in blue). Newly synthesized DNA strands annealing, driven by sequence homology, leads to the formation of four-way cruciform junctions known as reversed forks or *chicken feet*. Reversed forks can branch-migrate due to further positive supercoiling-driven parental strand re-annealing. Replisome components are represented as green and blue circles. The gray box delimits aberrant transitions leading to fork reversal. **(B)** Activation of checkpoint kinases counteracts gene gating. In S phase, replication forks engage RNA polymerase II-transcribed genes, which associate to the inner basket of NPCs through the action of co-transcriptional protein complexes and key nucleoporins (including Mlp1) in a process known as “gene gating.” Gated genes behave as barriers to topological stress diffusion as they counteract the rotation of helix strands around each other, thus favoring the accumulation of positive supercoiling ahead of approaching replication forks. Following replication stress Rad53 phosphorylates Mlp1 thus releasing transcribed genes from their association to the nuclear pores. Disengagement of transcribed genes permits DNA rotation and the diffusion of topological stress away from replication forks. In checkpoint deficient cells topological barriers persist, favoring positive supercoiling relaxation through reversal of stalled forks. Replisome components and the transcriptional apparatus are represented as green/blue and pink circles, respectively.

Collapsed replication forks in checkpoint deficient cells are characterized by the accumulation of abnormal replication intermediates (Lopes et al., 2001; Cotta-Ramusino et al., 2005). Prominently, checkpoint mutants exhibit forks in which nascent strands re-anneal to generate four-way junctions, often referred to as reversed forks (Sogo et al., 2002). Formation of reversed forks is promoted by the accumulation of torsional stress both *in vitro* and *in vivo* (Postow et al., 2001a,b; Bermejo et al., 2011; Ray Chaudhuri et al., 2012). Positive supercoiling generated by DNA unwinding at the replication fork tends to re-anneal parental DNA strands (Wang, 2002), thus regressing the fork branching point, and strip-off the nascent strands. Nascent strands can in turn pair due to their sequence homology (Figure 3A). Fork reversal driven by positive supercoiling is favored *in vitro* by protease treatments that eliminate replisome components from fork DNA, suggesting that the association of replisome factors with nascent DNA strands might counteract the topological transitions leading to fork reversal (Postow et al., 2001b). Recent evidence suggests that the replication checkpoint modulates chromosome architecture and can attenuate the impact of positive supercoiling on stalled forks (Bermejo et al., 2011; Dion et al., 2012; Mine-Hattab and Rothstein, 2012). Rad53 phosphorylates the Mlp1 nucleoporin, which mediates the association of RNA polymerase II transcribed genes to NPCs in a phenomenon known as gene gating (Kohler and Hurt, 2007). Transcribed chromatin associating with the fixed NPC structure would prevent the rotation of DNA strands around each other establishing a barrier to the diffusion of topological changes (Koster et al., 2010). In this view, positive supercoiling would tend to progressively accumulate as replication forks approach transcribed genes (Figure 3B). Upon treatment with HU, transcribed genes association with NPCs is released in a checkpoint-dependent manner (Bermejo et al., 2011), likely removing topological barriers that drive local positive supercoiling accumulation and promote fork reversal (Figure 3B). Reversed forks cannot sustain DNA synthesis and might represent a terminal step of fork stalling accounting for the loss of viability of checkpoint mutants upon treatment with replication stress inducing drugs. Genetic contexts suppressing fork reversal positively affect checkpoint deficient cells survival upon HU treatment (Bermejo et al., 2011) and mechanisms re-starting reversed forks in the absence of checkpoint kinases have not been described. It is, however, unclear whether fork reversal is necessarily a terminal event in eukaryotic cells (Ray Chaudhuri et al., 2012).

Nucleolytic processing activities also engage collapsed replication forks. Checkpoint mutants experiencing replication stress induced by dNTP pool depletion accumulate forks with extended ssDNA gaps and replication bubbles in which one of the nascent strands is absent (Sogo et al., 2002). The formation of gapped and hemireplicated molecules is partly dependent on the action of the Exo1 nuclease (Cotta-Ramusino et al., 2005). Ablation of Exo1 also reduces the accumulation of reversed forks (Cotta-Ramusino et al., 2005), suggesting that Exo1 might either promote their resolution by resecting reversed strands or the formation of extended ssDNA gaps precluding nascent strand re-annealing. Exo1 induces fork instability and lethality in checkpoint deficient cells that replicate damaged templates (Segurado and Diffley, 2008). Exo1 is phosphorylated by Rad53 (Smolka et al., 2007) and it has been suggested that targeting by Rad53 might downregulate Exo1 activity (Morin et al., 2008). This is in agreement with the notion that the checkpoint suppresses Exo1-mediated processing of normal and/or aberrant DNA structures at stalled replication forks, thus preventing fork breakdown (Segurado and Diffley, 2008). Exo1-dependent processing of collapsed forks is likely to prime unscheduled recombination events giving rise to gross chromosomal re-arrangements in checkpoint deficient cells (Myung and Kolodner, 2002; Kaochar et al., 2010). It has been proposed that further nucleolytic cleavage could target collapsed forks contributing to the formation of DNA breaks or as part of DNA repair attempts (Branzei and Foiani, 2009). The identity of the factors mediating such processing and the implication of the checkpoint in suppressing their action remain to be discovered.

Several replisome components including DNA polymerase α and δ subunits, as well as components of the Mcm2–7 and GINS helicase complexes are direct targets of Mec1 and Rad53 phosphorylation (Smolka et al., 2007; Chen et al., 2010; Randell et al., 2010) (Table 2) and the association of replicative polymerases and the Mcm2–7 helicase complex to stalled replication forks is impaired in checkpoint kinases mutants (Cobb et al., 2003, 2005; Lucca et al., 2004). These observations led to the suggestion that checkpoint kinases regulate the tethering of essential replisome components to fork DNA and that the loss of this tethering is the reason for checkpoint mutants inability to resume DNA synthesis. In agreement with this hypothesis, some replisome factors, such as the Mcm2–7 complex, cannot be re-loaded to replication forks (Labib et al., 2000), nor do efficient mechanisms for re-loading essential replication factors to collapsed forks seem to operate (Zegerman and Diffley, 2009). A recent study showed that the association between replisome components isolated by immunoprecipitation following genotoxic treatment is equivalent in wild type cells and checkpoint mutants (De Piccoli et al., 2012), suggesting that replisomes do not suffer gross structural alterations as a result of fork collapse. The same study showed that DNA polymerase α and Mcm2–7 complex components remained associated to a large fraction of replication forks following HU treatment in the absence of checkpoint kinases, raising the possibility that lack of phosphorylation of replisome components, rather than dissociation from replication forks, accounts for the inability of cells to re-start DNA synthesis (De Piccoli et al., 2012). Interestingly, forks from which replisome components are lost correlate with those emanated from earliest origins, though the specific determinants of the susceptibility of these forks to replisome dissociation are unclear.

**Figure 4:**
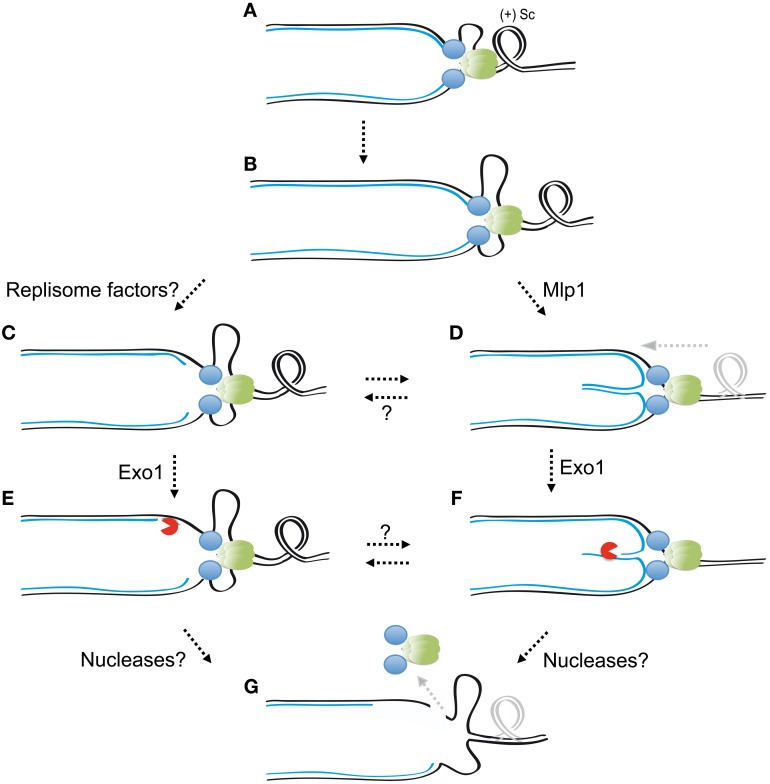
**Interplay between checkpoint-mediated mechanisms counteracting replication fork collapse.** In unperturbed conditions replication fork advancement generates positive supercoiling **(A)**. Fork stalling and helicase/polymerases uncoupling generate extended ssDNA tracks triggering checkpoint activation **(B)**. Replisome factors targeting by checkpoint kinases might limit replisome tracking and thus prevent the dissociation of DNA polymerases from nascent strands termini **(C)**. Checkpoint kinases also inhibit Mlp1 function, allowing positive supercoiling diffusion, and counteracting replication fork reversal **(D)**. In the absence of checkpoint kinases Exo1 could access exposed nascent strands termini and generate extended ssDNA tracks **(E,F)**. Further nucleolytic processing of ssDNA tracks or branch cleavage activities could determine the formation of DNA breaks, which may in turn favor replisome dissociation from fork DNA **(G)**. The combined action of these checkpoint-suppressed events likely contributes to the loss of functional integrity of stalled forks. Factors phosphorylated and potentially inhibited by checkpoint kinases to suppress abnormal fork transitions are indicated. Replisome components are represented as green and blue circles.

The relative contribution of replisome destabilization, replication fork reversal, and the nucleolytic processing of replication intermediates to the loss of replication fork functionality is unclear. It is likely that the three processes interplay to promote fork collapse if not effectively suppressed by checkpoint kinases (Figure 4). It is tantalizing to speculate that checkpoint kinases might somehow contribute to maintain the association of DNA polymerases with nascent DNA chains, perhaps by restraining helicase activity, or DNA polymerases processivity through direct phosphorylation events. DNA polymerases might be physically displaced from the 3′ termini of nascent strands by an excessive tracking of the replisome along the parental DNA, thus losing their capacity to continue DNA synthesis. The mechanical stress imposed by positive supercoiling may also contribute to displacing DNA polymerases from 3′ termini by peeling-off the nascent strands from the parental template. Nucleolytic processing at forks might in turn be favored by the exposure of the termini of nascent strands upon replisome dislodgement or fork reversal (Figure 4). Nucleolytic cleavage of ssDNA or branched structures could eventually generate discontinuities allowing the dissociation of replisome factors topologically linked to DNA (such as the Mcm2–7 complex or PCNA rings), thus accounting for the replisome loss observed in checkpoint mutants at early established replication forks (Cobb et al., 2003, 2005; Lucca et al., 2004; De Piccoli et al., 2012). Further work will be required to understand the checkpoint-mediated mechanisms protecting replication forks and their relative impact on genome integrity maintenance in response to different replication stress-inducing agents in detail.

## Checkpoint signaling reversion and restoration of normal cellular physiology

As discussed above, checkpoint activation in response to replication stress has a profound impact on several cellular processes, including modulation of the transcriptional program, replicon dynamics, and cell cycle progression. Checkpoint kinases phosphorylate and/or regulate the expression levels of a large number of factors. Once replication stress is overcome, normal cellular physiology needs to be restored. This requires shutting-off the checkpoint signaling cascade, as well as the reversion of posttranslational modifications and expression level changes of checkpoint transducers and effectors.

A first mechanism contributing to checkpoint signaling inactivation is likely to be the elimination of upstream signals recognized by checkpoint sensors at replication forks. Upon resumption of DNA synthesis, ssDNA tracks shorten as DNA polymerases catch up with each other or with helicases, thus limiting further Ddc2-mediated recruitment and activation of Mec1. Interruption of Mec1 signaling may be sufficient to shut-off the checkpoint response, as Mec1 activity downregulation by overexpression of a dominant negative *MEC1* allele results in pre-mature Rad53 dephosphorylation (Paciotti et al., 2001). However, an exclusively passive mechanism is unlikely to account for the rapid checkpoint inactivation observed after the removal of replication stress-inducing drugs (Travesa et al., 2008), indicating that mechanisms exist that actively revert checkpoint signaling.

A straightforward way to actively interrupt checkpoint signaling is to reverse the phosphorylation events mediated by checkpoint kinases. Work over the last years has focused on the inactivation of Rad53 kinase through dephosphorylation, which is quickly achieved upon the removal of replication stress inducing agents. Rad53 dephosphorylation tightly correlates with the downregulation of its kinase activity and does not require protein synthesis (Pellicioli et al., 1999). Budding yeast phosphatases Ptc2, Ptc3, and Pph3/Psy2 are required for Rad53 dephosphorylation following replication stress (O'Neill et al., 2007; Szyjka et al., 2008). The Pph3 phosphatase and its regulatory subunit Psy2 form a complex that dephosphorylates Rad53 *in vitro* and it has been suggested that they directly inactivate Rad53 (O'Neill et al., 2007). It is unclear, however, whether Pph3 has other important targets in checkpoint inactivation (Keogh et al., 2006). Ptc2 and Ptc3 are type 2C protein phosphatases with redundant functions in the checkpoint response (Leroy et al., 2003). Ptc2 and Ptc3 can bind Rad53 and directly mediate its dephosphorylation (Leroy et al., 2003; Guillemain et al., 2007). Both *ptc2*,* ptc3*, and *pph3*/*psy2* deletion mutants accumulate hyperphosphorylated Rad53 upon treatment with HU or MMS (Travesa et al., 2008). Ptc2/Ptc3 human homologs Wip1/PPM1D also play important roles in the reversal of DNA damage checkpoint responses by dephosphorylating and inactivating checkpoint components (for review see Heideker et al., 2007; Clemenson and Marsolier-Kergoat, 2009).

Interestingly, checkpoint inactivation has different genetic requirements upon continuous exposure to (adaptation) or during the recovery from replication stress. Pph3/Psy2 complex mediates Rad53 dephosphorylation both during adaptation and recovery from HU or MMS treatments, while Ptc2 and Ptc3 are dispensable during recovery (Travesa et al., 2008). The Glc7/protein phosphatase 1, recently shown to be involved in checkpoint inactivation, acts both during adaptation and recovery following HU treatment, but is dispensable during MMS exposure (Bazzi et al., 2010). These differential genetic requirements for checkpoint inactivation point to the existence of distinct modifications of checkpoint factors that might be crucial for checkpoint function in different cellular contexts or upon different replication stress-inducing stimuli. Recent observations indicate that checkpoint inactivation can also be achieved through degradation or cellular sorting of checkpoint transducers and/or effectors. Mammalian CHK1 effector kinase is inactivated through proteasome-dependent downregulation upon ATR-mediated phosphorylation, which leads to both CHK1 activation and its marking for degradation (Zhang et al., 2005). Furthermore, the Mrc1 human homolog CLASPIN is targeted for degradation in response to HU treatment, thereby promoting CHK1 inactivation (Mailand et al., 2006; Mamely et al., 2006; Peschiaroli et al., 2006).

Reversion of checkpoint-induced changes in the transcriptional program is promoted by the establishment of negative feedback loops (Smolka et al., 2012). As mentioned above, Crt1 and Nrm1 are phosphorylated by checkpoint kinases, which remove them from damage inducible and MBF targets gene promoters (Huang et al., 1998; Travesa et al., 2012). *CRT1* and *NRM1* promoters are bound by Crt1 and Nrm1, respectively, and therefore their transcription is upregulated along with that of other damage inducible genes upon checkpoint activation. Overexpression of Crt1 and Nrm1 provides a simple mechanism to limit checkpoint-mediated transcriptional changes, as accumulating Crt1 and Nrm1 might escape regulation by checkpoint kinases to mediate the repression of the relevant genes. This mechanism might be particularly efficient upon concomitant inactivation of checkpoint signal transduction, as newly synthesized Crt1 and Nrm1 would not be inhibited by checkpoint kinases.

Untimely persistence of checkpoint signaling might impact on replication dynamics. It has been suggested that checkpoint kinases slow down replication fork progression rates by directly phosphorylating replisome components (Labib and De Piccoli, 2011). Consistently, DNA synthesis resumption at MMS stalled forks is severely impaired in cells lacking Pph3 phosphatase (Szyjka et al., 2008). Such slowly progressing forks would need to traverse longer genomic regions before fusing, as persistent checkpoint signaling may also suppress late origin firing. Furthermore, the fidelity of DNA synthesis at these slow-progressing forks might be additionally compromised by the persistence of abnormally elevated dNTP pools. Therefore, a failure to promptly inactivate the checkpoint response once cells overcome replication stress might greatly impact genome integrity. In the future, it will be interesting to analyze which cellular mechanisms revert checkpoint signaling following different kinds of replication stress induced by diverse chemotherapeutic agents, as well as to study the impact of checkpoint inactivation defects on malignant transformation and cancer development.

## Checkpoint evolution as an anticancer barrier in multicellular organisms

Apical checkpoint kinases share homology with the PI3-related TOR kinases, which modulate cellular metabolism in response to nutrient availability (Lovejoy and Cortez, 2009). As mentioned above, budding yeast checkpoint kinases play an essential role in regulating dNTP pools even in the absence of replication stress (Zhao et al., 1998). It is therefore reasonable to think that the ancestral role of checkpoint kinases might have been to modulate the cellular metabolism in order to readily meet the elevated demand for dNTPs imposed by DNA replication during S phase. This function may have become crucial to ensure DNA replication fidelity in unicellular eukaryotes in which growth and proliferation greatly depend on nutrient availability (Alberghina et al., 2012). In this view, checkpoint kinases might have become progressively specialized in sensing and responding to stimuli requiring the upregulation of dNTP levels such as replication stalling by exogenous toxins or the repair of DNA damage. The evolutionary advantage of being able to survive such genotoxic insults might have favored checkpoint kinases gaining control over other cellular processes essential for replication integrity such as the control of cell cycle progression, replication origin firing, or replication fork stabilization. Checkpoint control of these functions is conserved in multicellular organisms (Jackson and Bartek, 2009; Ciccia and Elledge, 2010), although in higher eukaryotes the checkpoint response regulates mechanisms driving cells out of proliferating pools such as senescence or apoptosis. This might relate to the fact that cell proliferation decisions in higher eukaryotes are integrated at the organism level and are relatively independent from environmental nutrient availability.

In recent years it has emerged that the checkpoint response behaves as a barrier preventing tumorigenesis at early stages of cancer development (Bartek et al., 2007a; Halazonetis et al., 2008). This idea is supported by the observations that tumor cells, unlike other highly proliferating cells, show constitutively activated checkpoint kinases and markers of DNA breakage (Bartkova et al., 2005; Gorgoulis et al., 2005) and that oncogene activation induces replication stress, fork collapse, and formation of DNA breaks (Bartkova et al., 2006; Di Micco et al., 2006). Replication stress and DNA damage in this context may emerge from massive interference of replication forks with unscheduled oncogene-induced transcription (Bermejo et al., 2012b). In early pre-invasive lesions the checkpoint response is thought to promote pre-malignant cells removal from proliferating pools into senescence or apoptosis (Bartek et al., 2007b). Mutations or epigenetic silencing of checkpoint genes might result in an increased accumulation of DNA breaks owing to faulty fork stabilization, as well as to the loss of the checkpoint-mediated restraints to proliferation. Hence, cancer cells could proliferate and expand at the expense of an increased genomic instability, thus accelerating tumorigenesis.

Future research should focus on integrating the current insight on checkpoint-mediated replication fork protection with a deeper knowledge on the determinants driving fork collapse in cells experiencing oncogene-induced replication stress. It will also be interesting to explore the connections between checkpoint inactivation mechanisms, replication dynamics, and genome integrity maintenance; and how checkpoint signaling modulation might interplay with the checkpoint function in suppressing cell proliferation to act as an anticancer barrier.

### Conflict of interest statement

The authors declare that the research was conducted in the absence of any commercial or financial relationships that could be construed as a potential conflict of interest.
